# Using probabilistic genotypes in linkage analysis of polyploids

**DOI:** 10.1007/s00122-021-03834-x

**Published:** 2021-05-25

**Authors:** Yanlin Liao, Roeland E. Voorrips, Peter M. Bourke, Giorgio Tumino, Paul Arens, Richard G. F. Visser, Marinus J. M. Smulders, Chris Maliepaard

**Affiliations:** Wageningen University and Research Plant Breeding, P.O. Box 386, Wageningen, AJ 6700 The Netherlands

## Abstract

**Key message:**

In polyploids, linkage mapping is carried out using genotyping with discrete dosage scores. Here, we use probabilistic genotypes and we validate it for the construction of polyploid linkage maps.

**Abstract:**

Marker genotypes are generally called as discrete values: homozygous versus heterozygous in the case of diploids, or an integer allele dosage in the case of polyploids. Software for linkage map construction and/or QTL analysis usually relies on such discrete genotypes. However, it may not always be possible, or desirable, to assign definite values to genotype observations in the presence of uncertainty in the genotype calling. Here, we present an approach that uses probabilistic marker dosages for linkage map construction in polyploids. We compare our method to an approach based on discrete dosages, using simulated SNP array and sequence reads data with varying levels of data quality. We validate our approach using experimental data from a potato (*Solanum tuberosum* L.) SNP array applied to an F1 mapping population. In comparison to the approach based on discrete dosages, we mapped an additional 562 markers. All but three of these were mapped to the expected chromosome and marker position. For the remaining three markers, no physical position was known. The use of dosage probabilities is of particular relevance for map construction in polyploids using sequencing data, as these often result in a higher level of uncertainty regarding allele dosage.

**Supplementary Information:**

The online version contains supplementary material available at 10.1007/s00122-021-03834-x.

## Introduction

Polyploid species, including commercially important crops like potato, leek, rose, and chrysanthemum, have more than two copies of the genome. This makes the genetics of these species more complex than that of diploids and dedicated genetic analysis tools as well as much larger numbers of genetic markers are required. With the development of next-generation sequencing such datasets can now be generated. Meanwhile, a range of genetic analysis tools for polyploids has been developed (see the review of Bourke et al. 2018b). In most cases, raw genotyping data (e.g., fluorescence intensity ratios from single nucleotide polymorphism (SNP) arrays or read counts from sequencing data) are first processed to assign discrete genotypes to individuals; in the case of polyploid species, these processed genotypes are often in the form of marker allele dosage counts, or simply “dosages” (Bourke et al. 2018b). These dosages are then used as input for subsequent applications such as linkage map construction and quantitative trait locus (QTL) analysis.

However, discrete genotype calling may introduce errors and missing values in the data, resulting in loss of information (Tumino et al. 2016). In polyploid species, particularly those with higher ploidy levels (i.e., hexaploid and upwards), it becomes increasingly difficult to distinguish between heterozygous classes, especially if using sequencing data at lower read depth (Uitdewilligen et al. 2013). Therefore, downstream applications that can accommodate uncertainty in genotype calls (probabilistic genotypes) can carry more information through the analysis, leading to greater retention of markers and the possibility of increased genomic resolution. This may come at a cost however, as the discretizing of genotypes may also function as a marker filtering procedure.

Important downstream applications of genotype data are the construction of genetic linkage maps, QTL analysis, recombination mapping (identification of recombination hot and cold spots), or genome assembly, to name a few. This is especially true for polyploid species, many of which still lack a reference physical map. In autopolyploid species, linkage maps are usually developed using the F_1_ (or Full-Sib) progeny of a cross of two non-inbred, heterozygous parents. For polyploids, several software packages have been developed that perform linkage mapping, including TetraploidSNPMap (Hackett et al. 2017), PolyGembler (Zhou et al. 2020), polymapR (Bourke et al. 2018a), MAPpoly (Mollinari and Garcia 2019), and netgwas (Behrouzi et al. 2017).

**Fig. 1:**
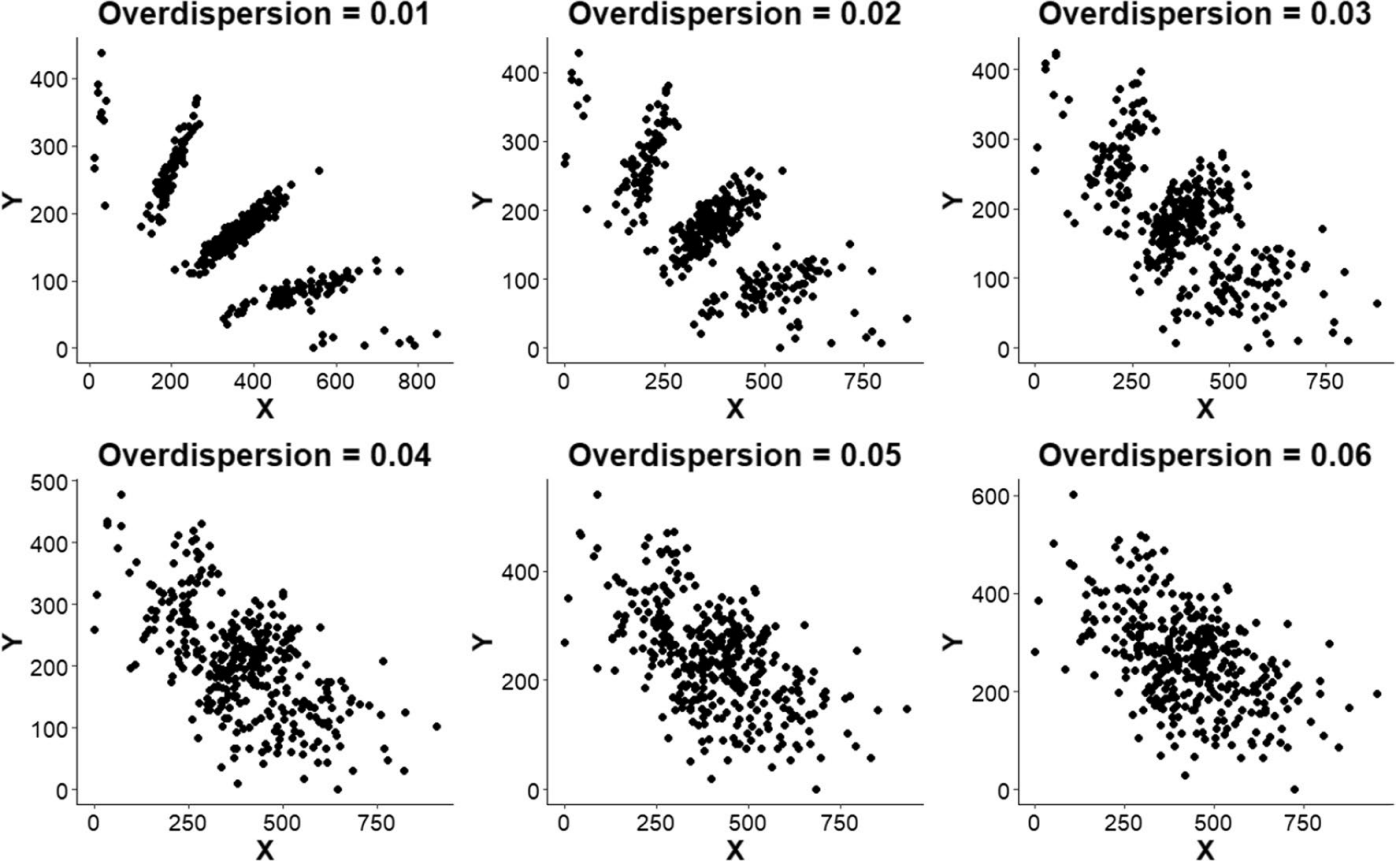
Examples of simulated SNP array data for a single SNP locus with different levels of overdispersion, for the same set of underlying SNP genotypes across a tetraploid F1 population. The X and Y dimensions represent the signal intensities for the two alleles of a SNP locus. In a tetraploid (as shown here), a maximum of five clusters is expected, representing five possible dosages of the counted SNP allele: 0 (nulliplex—N), 1 (simplex—S), 2 (duplex—D), 3 (triplex—T), and 4 (quadruplex—Q). Similarly, a maximum of seven dosage classes (0–6) is expected in a hexaploid

From the perspective of probabilistic genotypes, SNP array data are directly comparable to sequencing-based data from which bi-allelic SNPs have been called. Our method is applicable to both data types. Raw SNP array data consist of two signal intensities, corresponding to each of the two alleles. In a scatterplot of these SNP signal intensities, clusters are often visible, corresponding to the different dosage classes (Fig. 1**)**. When the clusters are compact and well separated, it is easy to distinguish these classes, resulting in high concordance between the true underlying genotype and the assigned genotype call. However, in cases where these clusters are not well-defined, genotype calling becomes problematic. A similar visualization can be generated for sequencing-based genotypes, with the numbers of reads for a particular allele replacing signal intensities on the axes (see for example Gerard et al. 2018).

Several software packages are available to convert array intensities to dosages in polyploids, including SuperMASSA (Serang et al. 2012), fitPoly (Voorrips et al. 2011; Zych et al. 2019) and ClusterCall (Schmitz et al. 2017). For sequence-based genotypes, specific polyploid-oriented software options include updog (Gerard et al. 2018) and polyRAD (Clark et al. 2019).

In genotype calling of sequence reads, the quality of the call is related to read depth (Gerard et al. 2018; Matias et al. 2019; Uitdewilligen et al. 2013; Yamamoto et al. 2020). A read depth of 60× to 80× was recommended by Uitdewilligen et al. (2013) to obtain high-quality dosage estimates in an autotetraploid. Gerard et al. (2018) suggested read depths of 25× and 90× for autotetraploids and autohexaploids, respectively. In experimental studies mean read depths of 41×, 63×, and 44× were applied in triploid banana (Davey et al. 2013), autotetraploid potato (Uitdewilligen et al. 2013), and hexaploid sweetpotato (Yamamoto et al. 2020). In those studies, discrete dosages were generated. Here, we investigated whether the use of probabilistic genotypes could be helpful in situations where read depth is moderately low. In practice, the recommended read depths are not always achieved. Indeed, a balance must be found between accurate genotyping (high depths) and cost-effectiveness (lower depths).

Here, we investigate the use of dosage probabilities instead of discrete dosages in the context of linkage mapping in autopolyploid F1 populations. We used simulation studies to compare the results of linkage estimation based on both discrete and probabilistic dosage data from SNP array and sequence reads. We also performed simulations to study the effect of read depth on the quality of genotype calling and subsequent linkage analysis. Furthermore, we validated our approach by re-analyzing an experimental tetraploid potato dataset, comparing our results with those from a previously published high-density integrated map that was based on discrete dosage scores obtained from the same array data (Bourke et al. 2016).

## Materials and methods

### Genotype calling

Genotype calling was performed on simulated SNP array and sequence read data and on actual potato SNP array data, as described below. Simulated and real SNP array data were processed with fitPoly (Voorrips, et al. 2011; Zych et al. 2019), and simulated sequence read data were processed with updog (Gerard et al. 2018) to obtain discrete dosages and dosage probabilities. In both platforms, mixture models are fitted to the signal intensity ratios or sequence read counts. This results in a set of probabilities, summing to 1, of each of the (ploidy + 1) dosage classes of the alternative allele of a marker, per individual. From these dosage probabilities, discrete dosage scores are obtained by assigning the dosage with the maximum probability, if that is above a user-defined threshold (e.g., 0.95). A missing value is assigned if all probabilities are below the threshold. A marker is discarded when the proportion of individuals with missing dosages is above another user-defined threshold (0.4). As a consequence, potentially large numbers of markers may be removed. In our study we directly used the probabilities and omitted the filtering for the dosage probabilities and minimum fraction of scored individuals, thereby retaining all available information.

### Data filtering

A number of pre-mapping steps are included in polymapR (Bourke et al. 2018a) which we have adopted here and generalized for probabilistic genotypes. These include the identification and removal of duplicate individuals, poorly scored individuals, or markers with unclear segregation types. Note that discrete dosage data can include missing values, while probabilistic dosages do not (even if individuals and markers with low-quality data are removed, the remaining dataset has no missing values).

#### Duplicate individuals

In the polymapR pipeline, duplicate individuals are currently detected by calculating the Pearson correlation coefficients between all pairs of individuals over all marker scores. For discrete dosages this is straightforward; using probabilistic dosages, we define the weighted marker dosage *X*_*ij*_ of the *i*th marker in the *j*th individual as:

$$X_{ij} = \mathop \sum \limits_{n = 0}^{c} n \cdot P\left( {X_{ij} = n} \right)$$

where *c* is the ploidy. All pairs of individuals with a correlation coefficient higher than 0.95 were assumed to be duplicates and merged, where merging consists of taking the average of the probabilities for each dosage class over the duplicate individuals.

#### Poorly scored individuals

Some individuals may be poorly scored overall, perhaps due to low DNA quality or deviating ploidy; these individuals should be identified and omitted from the analysis. We identified problematic individuals as those where fewer than half of their markers had a probability greater than 0.6 for one of the dosage classes.

#### Marker segregation type

In an autopolyploid F_1_ cross progeny, the combination of the parental dosage scores defines the marker segregation type (Supplementary Table 1). For instance, the notation ‘SN marker’ refers to a Simplex × Nulliplex marker with dosage 1 (Simplex, S) in the first parent and dosage 0 (Nulliplex, N) in the second parent and an expected 1:1 (nulliplex: simplex) segregation in the progeny. For each marker we tested whether the (probabilistic) segregation observed in the progeny matches the segregation type defined by the most probable dosages of the parents. More details about the determination of marker segregation types are included in Appendix 1.

### Linkage estimation

Before estimation of recombination frequencies, marker dosages are converted to their simplest form in the polymapR pipeline to simplify the linkage analysis (Bourke et al. 2016). This method was adapted to accommodate probabilistic dosages.

The two-point linkage analyses rely on the method of maximum likelihood to estimate recombination frequency (*r*) assuming bivalent pairing. In Bourke et al. (2018a), the observed counts of individuals in each class $O\left( {n_{ij} } \right)$ and the expected frequency of each offspring class $E\left( {n_{ij} } \right)$, where *n*_*ij*_ is the number of individuals with dosages *i* and *j* at the two loci, together yield the likelihood function (with *n* = ploidy/2):

1 $$L\left( r \right) \propto \mathop \prod \limits_{i,j = 0}^{2n} E\left( {n_{ij} } \right)^{{O\left( {n_{ij} } \right)}}$$

With probabilistic genotypes, the full likelihood equation must be employed:

2 $$L\left( r \right) \propto \mathop \prod \limits_{z = 1}^{{n\;{\text{ind}}}} \mathop \sum \limits_{i,j = 0}^{2n} p_{ij,z} E\left( {n_{ij} } \right)$$

where $p_{ij,z}$ is the probability of genotype (*i*, *j*) for individual *z* (Mollinari and Garcia 2019). In cases where each individual is assigned a discrete genotype (i.e., one dosage class’ probability is one and all other classes are zero), formula (2) simplifies to formula (1). In our study, the linkage estimation was done with MAPpoly using an implementation of Eq. (2). Later, the output was converted to be compatible with polymapR for further constructing of the linkage groups.

### Ordering

The ordering of the markers was done with MDSMap (Preedy and Hackett 2016). After the first ordering, the nearest neighbor fit (NNfit, the absolute difference between the observed and estimated distance of a marker to its nearest informative neighbor) was used to remove outliers (NNfit > 5) and ordering was performed again for the remaining markers (Preedy and Hackett 2016) to minimize the effect of deviating markers.

### Simulation of SNP array genotyping data

#### Simulated datasets

The simulation software PedigreeSim (Voorrips and Maliepaard 2012) was used to generate datasets of biparental F_1_ populations. We simulated 200 F_1_ individuals of a fully autotetraploid species with 1 chromosome of 100 cM and the centromere in the middle. SN, SS, SD, DN, and DD markers (Supplementary Table 1) were simulated at 1 cM spacings, resulting in 505 markers over the chromosome. One hundred such datasets were simulated. Based on the known (simulated) allele dosages, an in-house script (Supplementary file 2) was used to simulate SNP array signal intensities for all F1 individuals and both parents with six different settings of an “overdispersion” parameter (ranging from 0.01 to 0.06; Fig. 1). Overdispersion (Gerard et al. 2018) was used to describe additional variability relative to the expected variation under a basic model. Details about the simulation parameters are described in Appendix 2. Dosage probabilities were then estimated by fitting mixture models using fitPoly (an extension of the fitTetra package Voorrips et al. 2011; Zych et al. 2019) available from CRAN).

#### Linkage analysis with simulated datasets

The simulated data was used for linkage estimation using two approaches*: ‘Probabilistic’* (the probabilities of all five possible marker dosages as estimated by fitPoly) and *‘Discrete’* (the discrete dosage with the highest probability, but the dosage replaced by a missing value if this highest probability was less than 0.95). For both approaches we followed the same workflow; only the linkage estimation function was different. For the *‘Discrete’* and the *‘Probabilistic’* approaches, the likelihood functions (1) and (2) were used, respectively.

The fraction of markers for which fitPoly was able to fit a mixture model differed between different levels of overdispersion. To investigate the marker quality in our simulated datasets, we looked at the average maximum genotype probabilities among all loci for all individuals. We compared the marker segregation type expected from the parental dosage scores and the segregation type best fitting the offspring data across the two sets of data; non-fitting markers were filtered out (as mentioned in ‘Marker Segregation Type’). An example of the difference between *‘Probabilistic’* and *‘Discrete’* in the determination of the marker segregation is shown in Appendix 3**.** To examine the results of the marker segregation determination, we evaluated the number of markers retained after filtering, and the fraction of the retained markers assigned an incorrect segregation type.

Before performing linkage analysis, we followed the data curation workflow described by Bourke et al. (2016). Under different levels of overdispersion, different numbers of markers were accepted for mapping. Because we applied a threshold (0.1) for the maximum allowed fraction of missing data per marker, fewer markers were retained for *‘Discrete’* than for *‘Probabilistic’*.

After the evaluation of the assignment of segregation type, we compared the accuracy of phasing and recombination frequency estimation between datasets. The fraction of incorrect pairwise marker phases was obtained by comparing the estimated and true (=simulated) phasings. Marker ordering was performed as described using MDSMap. Afterward, we examined how many markers were mapped by the two approaches and evaluated their estimated positions.

### Simulation of sequence reads genotyping data

Based on the same population settings that we used for the simulated SNP array data (biparental F1 population of 200 individuals, fully autotetraploid species, one chromosome with 100 cM length, centromere in the middle, and all possible marker types at 1 cM spacings, totally 505 markers), dosages were simulated by PedigreeSim for 10 populations. Based on these simulated dosages, read counts were generated by updog. The read depth varied between data points from 50× to 200×, the sequencing error rate was fixed at 0.001 and the allelic bias at 0.7 for all markers (reference/alternative). Overdispersion ranged 0.01–0.06. Once sequence read counts were generated, updog was used for genotype calling. The probabilistic dosages obtained from updog were processed as the array-derived dosage probabilities, using the pipeline described above. The simulated datasets were then processed through the pipelines of the *‘Probabilistic’* and *‘Discrete’* approaches, and the results were compared.

### Effect of read depth on the mean probability of correctly assigned dosages

Based on the same population settings as were used for the simulated sequence reads data, we simulated a wider range of fixed read depths, 10×, 20×, 40×, 60×, 80×, 100×, and 120× coverage per individual to investigate the effect of read depth on dosage calling; overdispersion levels of 0, 0.06, and 0.13 were used. Coverages of 10× and 20×, lower than the minimum read depths (25×) suggested by the authors of updog (Gerard et al. 2018), were used to explore the behavior in low-depth situations. Coverages of 40×, 60×, and 80× were close to reported depths in autotetraploid studies. Coverages of 100× and above, considered high read depth, were used assuming that these would result in high-quality dosage calls by updog.

### Effect of thresholds on discrete dosage calling

To understand whether any advantage accrues from the use of probabilistic dosages, we investigated the effects of different thresholds on the number of markers retained, as well as the consequences for linkage analysis. Based on the results of the simulations to study the effect of read depth, we used probability thresholds of 0.85, 0.9, and 0.95 for assigning a dosage score, and thresholds of 0.05, 0.1, 0.15, and 0.2 for the fraction of missing dosage scores that resulted in rejecting a marker.

### Re-analysis of an experimental potato dataset

A potato SNP array dataset that had previously been used for autotetraploid linkage map development (Bourke et al. 2016) was re-analyzed to evaluate the use of dosage probabilities in experimental data. This dataset consists of an F_1_ population of 237 individuals from a cross between two tetraploid cultivars (*Altus* × *Colomba*), with 17,987 SNPs genotyped using the SolSTW Infinium SNP array (Vos et al. 2015). The output of fitPoly was analyzed using the *‘Probabilistic’* approach as described in the previous sections. The results of this approach were compared with published results which used the *‘Discrete’* approach as described in the previous section.

In this study, we constructed both an integrated map and maps of all homologs separately. The integrated map combines all markers per linkage group from all homologous chromosomes across both parents. The homolog maps were based on the recombination frequencies among only the markers tagging each separate homolog. Linkage mapping was performed for dosage probabilities and included the major steps: linkage estimation, clustering, ordering, and checking of the map quality. A detailed description of the steps in map construction is given in Bourke et al. (2016) followed by marker ordering (as described in ‘Ordering’). Marker pairs with a Pearson correlation coefficient higher than 0.99 were treated as identical. For each set of identical markers, only the one with the largest number of significant linkages to other markers in the same linkage group was retained as the representative marker. After map construction, the identical markers that were previously set aside were assigned to the same position and homolog (s) as the representative mapped marker of their set.

After construction of the *‘Probabilistic’* map we compared it with the published map. Both linkage maps were aligned with the potato reference genome v4.03 (Potato Genome Sequencing Consortium 2011). The integrated *‘Probabilistic’* map was also aligned with the homolog maps to check the consistency of the map integration. To quantify the consistency between the *‘Probabilistic’* map and the published map, we used the positions of the markers appearing on both maps to fit a linear regression, where we calculated the adjusted *R*^2^ and slope for each homolog. To check whether additional markers in the *‘Probabilistic’* map that did not appear in the published map have credible positions, we BLASTed the sequences of all additional markers to the potato reference genome v4.03 to check their putative physical location. We also calculated the average maximum probability (over individuals) of these additional markers and compared these values with those of the markers in the published linkage map.

## Results

### Simulation of SNP array genotyping data

#### Effect of overdispersion on dosage probabilities

Dosage classes can be distinguished better if the clusters of allele signal ratios of individuals corresponding to different dosages are well separated. With increasing overdispersion, the probability of the most probable dosage classes (averaged over all individuals) decreases.

#### Accuracy of marker segregation determination

The expected segregation in the F1 population is determined from the most probable dosages of the parents. At higher levels of overdispersion more parental dosages became missing, resulting in fewer markers being retained for both genotyping approaches. When overdispersion was relatively low (≤ 0.03), there were only minimal differences between these two methods. When overdispersion was higher than 0.03, no markers were kept in the *‘Discrete’* approach due to a high rate of missing values (threshold for the fraction missing values: 0.05). At overdispersion of 0.04, 48% of the markers were retained in the *‘Probabilistic’* approach; for the other 52% at least one of the parental dosages was missing. Ten percent of the retained markers was assigned an incorrect segregation type. This mainly involved: (a) DN markers incorrectly identified as TS markers (45%); (b) DD markers incorrectly identified as SS markers and vice versa (41%); (c) SD markers identified as TN markers (10%). In all these cases the true segregation (a: 1:4:1; b: 1:8:18:8:1; c: 1:4:4:1) was similar to the incorrectly assigned segregation (a and b: 1:2:1; c: 1:1).

#### Accuracy of recombination and phase estimation

**Fig. 2:**
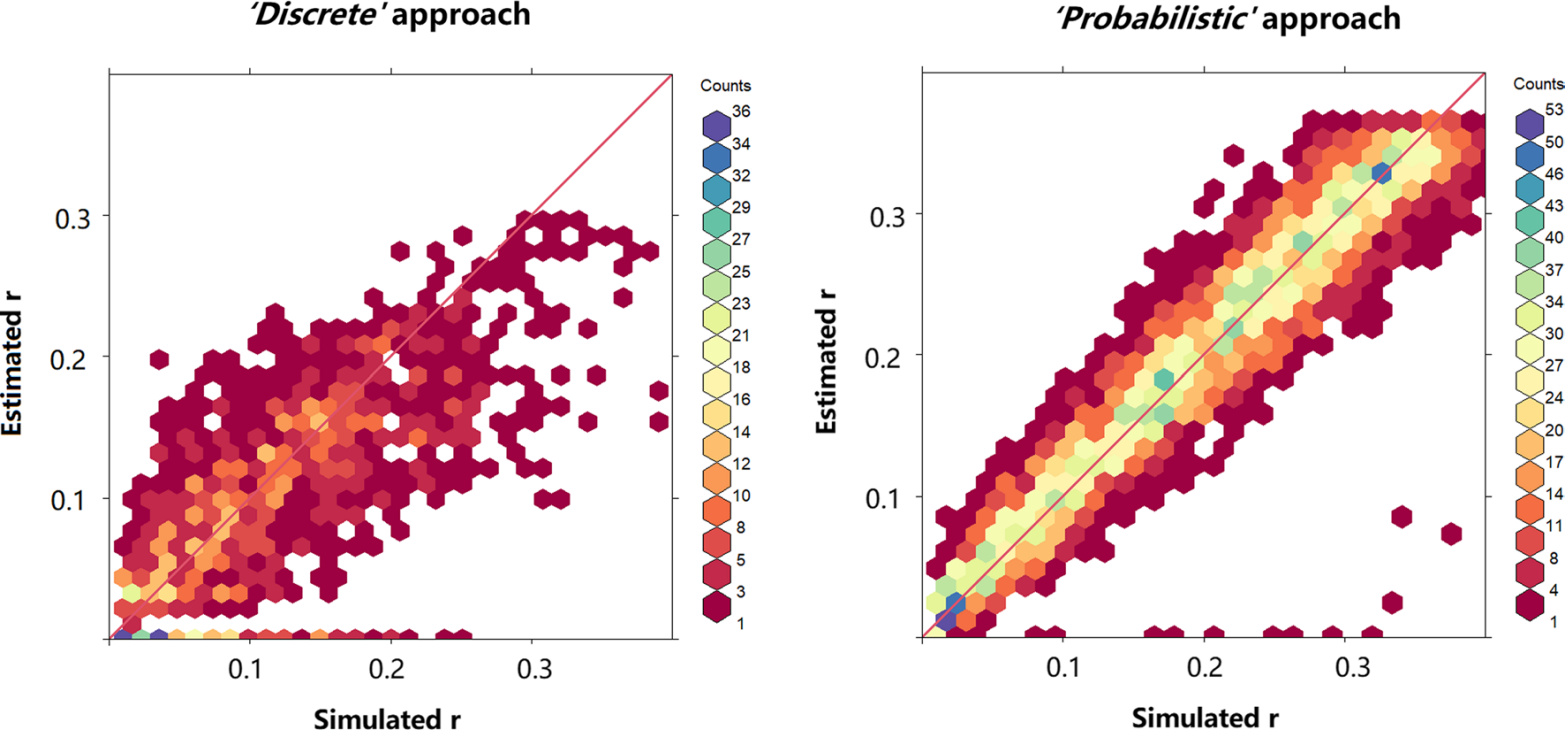
Estimated vs. true (simulated) recombination frequencies (*r*) at an overdispersion of 0.03 for pairs of SN-segregating markers in coupling phase. The red line shows *y* =  *x*

After the segregation check, the number of markers retained with *‘Probabilistic’* was larger than with *‘Discrete’* and the difference increased with increasing overdispersion. The same was true for the number of marker pairs available for recombination frequency estimation: at an overdispersion of 0.04 and higher, no marker pairs were left for *‘Discrete’*. At lower levels of overdispersion (< 0.03), no differences were observed in accuracy of the recombination frequency and phasing estimate between the two methods At an overdispersion of 0.03, an average of 39 individuals were retained for linkage estimation in the *‘Discrete’* due to the large amount of missing values per individual; in the *‘Probabilistic’* case all 200 individuals were retained. Therefore, the recombination frequency estimation of *‘Probabilistic’* approach outperformed the *‘Discrete’* approach (Fig. 2). The phase estimation of *‘Discrete’* was slightly better than for *‘Probabilistic’* (up to 4% less incorrectly phased marker pairs) when overdispersion was below 0.04. This was due to the fact that for some of the marker pairs that were incorrectly phased with the *‘Probabilistic’* method, one or both markers were rejected in *‘Discrete’*. At higher levels of overdispersion (> 0.03), the comparison was not possible as there were no marker pairs left for *‘Discrete’* (an example of SN-SN pairs is given in Supplementary Figures 1 and 2).

#### Accuracy of marker positions

After establishing the marker order, estimated marker positions were compared with the simulated positions. For lower values of overdispersion (< 0.03), 354 markers or more (out of 505) were mapped with both methods and their estimated and simulated positions agreed well (in regression of the estimated vs. simulated positions the slope was 1.05, the adjusted *R*^2^: 0.95)*.* At an overdispersion of 0.03, on average 156 and 304 markers mapped with the *‘Discrete’* and *‘Probabilistic’* approaches, respectively. For *‘Discrete’*, the estimated positions were inaccurate (slope: 1.6; adjusted *R*^2^: 0.32), while *‘Probabilistic’* still gave good estimates (slope: 1.09; adjusted *R*^2^: 0.94). For overdispersion above 0.04, no marker pairs were retained for ‘*Discrete*’ and therefore the marker order and positions could not be studied. For *‘Probabilistic’*, at an overdispersion of 0.04, on average 197 markers were mapped (slope: 0.99; adjusted *R*^2^ 0.71). Markers with an incorrect assigned segregation type were always removed at the mapping stage because of not fitting well with neighboring markers. The effect of incorrect phasing due to an incorrect segregation type therefore was minimal. At overdispersion > 0.04, few markers (less than 50) were retained also with ‘Probabilistic’ and their mapped positions did not show a clear linear relationship with the true positions in our simulation.

### Simulation of sequence reads genotyping data

The analysis of simulated sequence reads data showed similar results to the SNP array simulations: more markers were retained for analysis using the *‘Probabilistic’* approach. At overdispersion of 0.01, the segregation type of all markers was correctly assigned for both approaches. In linkage estimation, no markers were incorrectly phased. Both the *‘Probabilistic’* and the *‘Discrete’* approach gave high accuracy of recombination frequency under different levels of overdispersion. We fitted a regression model of estimated *r* on simulated *r*. For *‘Probabilistic’* and ‘*Discrete*’ approach, the slope and adjusted *R*^2^ were 0.95 vs. 0.96 and 0.78 vs. 0.78, respectively. In the map ordering, the *‘Probabilistic’* approach gave slightly better results than the *‘Discrete’* approach from the regression of estimated vs. simulated position (slope: 1.11 vs. 1.16; adjusted *R*^2^: 0.99 vs. 0.99). With overdispersion above 0.01, no markers were kept for analysis in *‘Discrete’* approach. In the *‘Probabilistic’* approach, at overdispersion between 0.02 and 0.06, at least 400 of the 505 markers were retained. When fitting the regression model of estimated r and simulated r, the slope was between 1.04 and 1.1 and adjusted *R*^2^ was 0.99 over all simulated overdispersion levels. (The results of marker segregation, linkage estimation, and map ordering evaluation are given in Supplementary Figures 3, 4, 5.)

### Effect of read depth on the mean probability of correctly assigned dosages

**Fig. 3:**
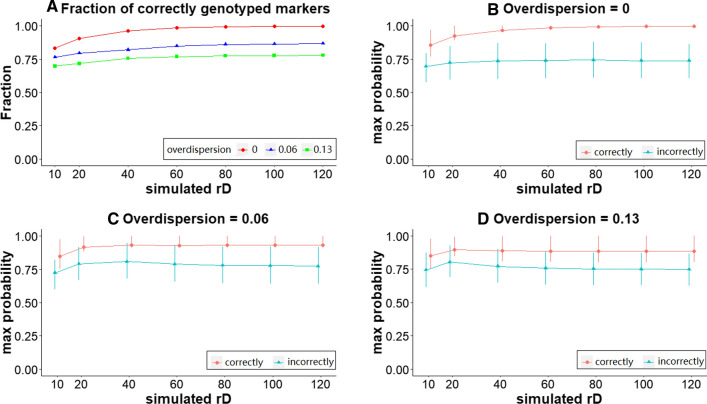
Comparison of correctly and incorrectly assigned genotypes at various levels of overdispersion (0, 0.06, and 0.13), and different read depths (rD). **a** The fraction of correctly genotyped markers (when discrete dosages are used). **b**–**d** The probability of the most probable dosage (max probability): the mean is indicated by dots; the ends of the bars represent the 25% and 75% quantiles (the bars are not observable for the upper left figure because the ranges are very small)

The fraction of correctly assigned dosages under the *‘Discrete’* approach and the probability scores with different overdispersion and read depth values are shown in Fig. 3.

*Fraction correctly assigned dosages:* Lower read depths led to more incorrect genotype calling. Without overdispersion, 83%–91% of the assigned dosages were correct at low read depths (10×, 20×), and 96%–99.2% at higher read depths (40×, 60×, 80×). At very high read depths (100×, 120×), more than 99.6% of the assigned dosages were correct. With overdispersion 0.06 and 0.13, 82.1% and 75.6% of the assigned dosages were correct, respectively, when read depth was above 40× (Fig. 3A).

*Probability scores:* With increasing overdispersion, the maximum probabilities of correctly and incorrectly assigned dosages become closer (no threshold used for filtering). Without overdispersion, at low read depths (10×, 20×) the mean maximum probability of correctly assigned dosages was 0.89, while that of incorrectly assigned dosages was 0.7 (Fig. 3B). This suggests that a threshold of 0.8 could help in limiting the number of genotyping errors under these conditions. When read depth was higher than 40×, the mean maximum probability for incorrect dosages was 0.74, while for correct dosages this was 0.98. For higher read depths therefore, a threshold of 0.85 or higher could be used. At overdispersion of 0.06, the difference of the mean maximum probability between incorrectly (0.77) and correctly (0.9) assigned dosages was smaller at read depth >=40× (Fig. 3C), and even more so at an overdispersion of 0.13 (0.75 and 0.88, respectively; Fig. 3D). This shows that is not straightforward to set a threshold for the maximum probability, and the result will depend not only on the (known) read depth but also on the (unknown) level of overdispersion.

### Effect of thresholds on discrete dosage calling

In the ‘Discrete’ case, different thresholds for the maximum dosage probability and for the missing value rate in the subsequent marker filtering are expected to lead to different numbers of markers retained for mapping. The results from the simulation study confirmed this. An example of how many markers are retained in the ‘Discrete’ case under different threshold values when overdispersion is zero is shown in Supplementary Figure 6. With a read depth of 40× (representative for a number of previous experimental studies, listed above) 62.5% of the markers were retained with a minimum probability threshold of 85% and a maximum missing value rate of 0.15. In contrast, in the ‘Probabilistic’ case no markers are filtered out, resulting in almost 40% more markers being retained for mapping.

### Re-analysis of the experimental potato dataset

#### Data filtering

We constructed a potato linkage map using the *‘Probabilistic’* approach, starting with results from the same fitTetra analysis of array data as Bourke et al. (2016). After fitting the F1 segregation, with dosage probabilities more markers were retained than in the published map. All markers available for mapping in both cases had the same estimates for the segregation type. After removing monomorphic (non-segregating) markers and markers with more than 10% missing values, 6912 markers (38.4%) were used for linkage analysis in the case of the published map based on discrete dosages (Bourke et al. 2016). Because no markers were rejected due to missing values for the *‘Probabilistic’* approach, all 7487 segregating markers were available for mapping (Table 1**)**.

**Table 1:** Marker filtering steps in the published linkage study and in the ‘Probabilistic’ approach

Steps in SNP filtering	Number of markers			
	Bourke et al. (2016)		*‘Probabilistic’*	
Total SNPs on array	17,987		17,987	
Probabilities and dosages assigned by fitTetra	15,266	84.9%	15,266	84.9%
F_1_ pattern acceptable*	13,774	76.6%	14,003	77.8%
Monomorphic	6558	36.5%	6516	36.2%
Polymorphic	7216	40.1%	7487	41.6%
Polymorphic and ≤ 10% missing	6912	38.4%	–	
Available for mapping	6912		7487	

*For the marker segregation type determination, Bourke et al. (2016) used discrete dosages, while *‘Probabilistic’* uses dosage probabilities as returned by fitTetra.

#### Map construction

Based on the dosage probabilities approach outlined in the *M&M* section, we constructed both an integrated map and maps of all individual homologs. The integrated map (*‘Probabilistic’* map) was compared with the published integrated map (Bourke et al. 2016) that was based on the same array data. The *‘Probabilistic’* map and published map both cover all 12 potato chromosomes. The published map has a total map length of 1061 cM and includes 6902 markers. The *‘Probabilistic’* map is 1082 cM long and includes 7244 markers. The two maps have 6682 markers in common.

The published map contains 228 markers over different chromosomes that are not on the *‘Probabilistic’* map. Of these, 23 were filtered out in the *‘Probabilistic’* approach because the segregation type did not match between the assignment by parental dosages and the assignment by the observed segregation in the F1 progeny. A total of 140 markers were filtered out due to the weak linkage (LOD < 3) with SN markers in the marker assignment step. The other 75 markers were filtered out in the marker ordering step, because they did not fit well with neighboring markers (nearest neighbor fit (NNfit) > 5 cM in MDSMap).

**Fig. 4:**
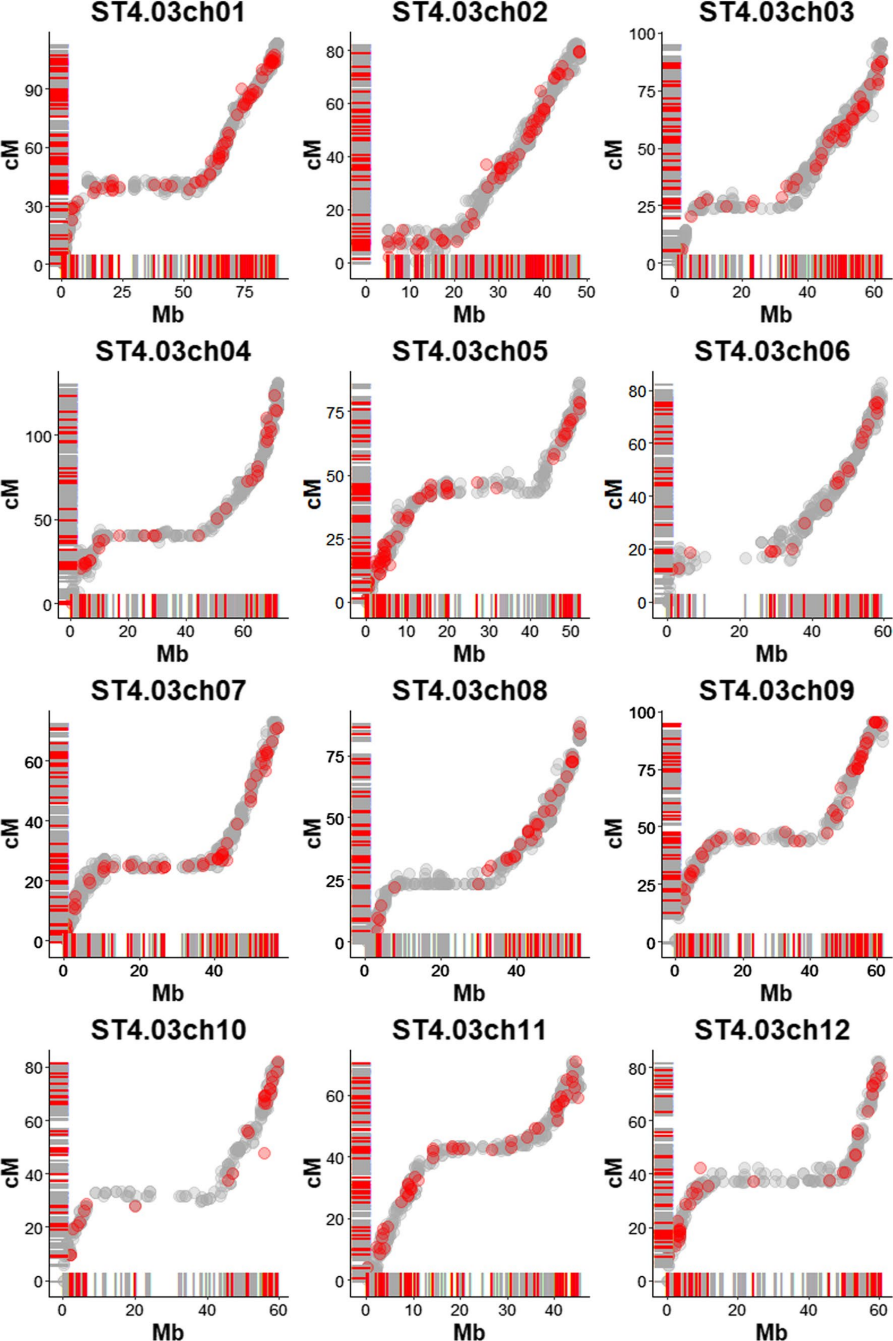
The positions of markers on the *‘Probabilistic’* map plotted against their positions on the reference genome: gray dots represent the markers on the published map, and red dots represent the 559 additional markers mapped only on the ‘Probabilistic’ map. The segregation types and genome positions of the additional markers are provided in Supplementary Fig. 6

On the other hand, the *‘Probabilistic’* map contains 562 markers that are not on the published map (locations and segregation types of these additional markers are provided in Supplementary Figure 7). The average maximum genotype probability over all markers on the published potato map was 0.995, compared to an average of 0.963 for these additional 562 markers. Physical map positions of 559 out of the 562 additional markers were available and corresponded well with their genetic map positions (Fig. 4).

#### Map quality

**Fig. 5:**
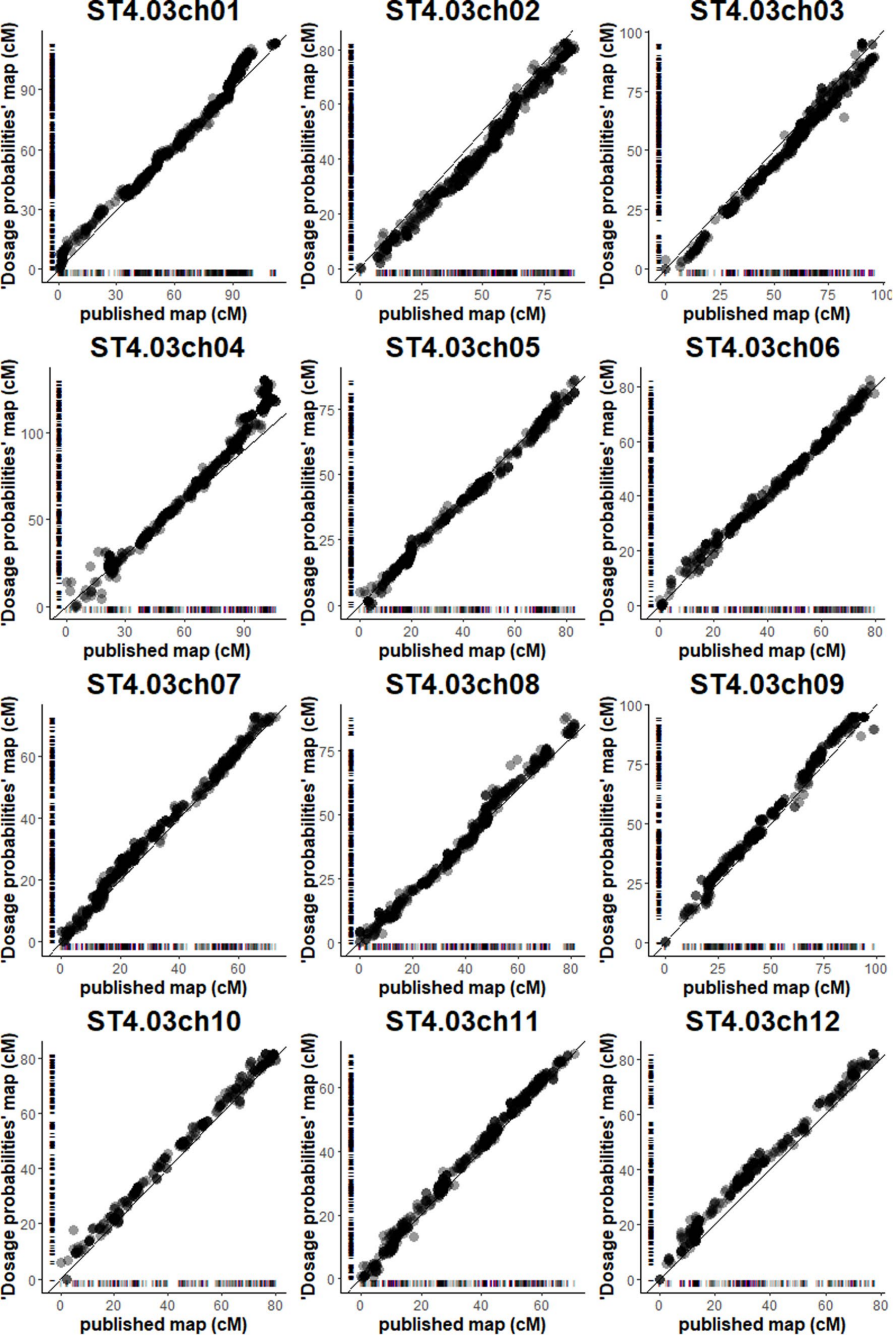
Comparison of the published map of Bourke et al. (2016) with the *‘Probabilistic’* map, per chromosome. The little stripes along the *x*- and * y*-axis indicate marker positions

To check the concordance of the *‘Probabilistic’* map with the reference genome, the maps were aligned per linkage group (Fig. 4). High consistency between *‘Probabilistic’* map and the published map was observed (Fig. 5). The maps of the individual homologs constructed from the dosage probabilities were also aligned to the integrated *‘Probabilistic’* map to check the quality of the integration across homologs. Here also a high consistency between the integrated map and the individual homolog maps was observed (Supplementary Figure 8).

## Discussion

### The use of dosage probabilities

In genotyping of polyploids, genotypes may either be assigned as discrete dosages, or as probabilities of a range of possible dosages. The latter type is arguably more informative—having no missing values as well as providing information on the level of uncertainty in a particular estimated genotype call. When two or more dosage classes have elevated probabilities, and therefore all are below a threshold of, say 0.90 or 0.95, it is not possible to assign a reliable discrete dosage score. The missing values introduced by this filtering lead to rejection of markers in a subsequent filtering based on missing value rate. Setting thresholds in filtering ensures the quality of markers retained for linkage analysis. However, strict thresholds also lead to the loss of usable markers. Lowering this threshold on the maximum probabilities has the effect of retaining more markers, but it also may also lead to more erroneous discrete dosage scores. However, the probabilities themselves still provide information about the possible dosages and this can be used in linkage analysis as demonstrated in this study. Probabilistic marker dosages enable these genotype uncertainties to be taken into consideration and carried through a linkage analysis, allowing more marker information to be used for downstream applications, such as genetic mapping and QTL analysis. In the high-quality potato dataset used here, using the dosage probabilities allowed for 577 (3%) more markers to be kept for linkage analysis.

Most of the steps in the probabilistic approach (*‘Probabilistic’*), are similar to the approaches using discrete dosages, including the determination of segregation type, which is now based on sums of probabilities instead of discrete counts per dosage class. In both cases markers showing a mismatch between the observed segregation in the F1 and that expected from the parental genotypes are discarded. In simulations with overdispersion, the use of dosage probabilities resulted in a slightly larger fraction of markers with an incorrect assigned segregation type. For the ‘*Discrete*’ approach, these markers were mostly rejected due to an excess of missing values. For the ‘*Probabilistic*’ approach, markers with an incorrect segregation type mostly result in incorrect phasing estimates and subsequently still drop out in the mapping process and therefore do not affect the quality of the final map. In the experimental potato dataset, for all but 23 markers the estimated segregation types were identical between two approaches.

In our simulation study with both SNP array and sequence reads data, more markers were retained for mapping with the *‘Probabilistic’* approach than with the ‘*Discrete*’ approach. These additional markers fitted well in the linkage maps, and their estimated positions corresponded well with the simulated positions. In the experimental potato dataset, an additional 562 markers were mapped. Of these, 559 aligned well with the potato reference genome (the putative physical chromosome and position); the remaining three had no known physical position. The map length of the *‘Probabilistic’* map is slightly higher (1082 cM, vs. 1061 cM for the published map); however, the two map lengths are not directly comparable because they use a different marker ordering algorithm: here we used MDSMap (Preedy and Hackett 2016) whereas the published map order was calculated using JoinMap® (Van Ooijen 2006).

We show that the use of probabilistic dosage data produces results that are at least as good as discrete dosages with a high-quality data set. Also, probabilistic data allows the use of markers of lower quality (higher overdispersion) where the signal ratios are concerned. However, our simulation study showed that in a scenario where all markers have high levels of overdispersion, very few markers are retained in the map even when using the *‘Probabilistic’* approach, so the issue of letting pass low-quality markers is moot. In our simulations of SNP array data, at intermediate overdispersion values (0.03 to 0.04), the *‘Probabilistic’* approach retained more markers that have the potential to be mapped, while in the *‘Discrete’* approach none of these markers were retained. With the simulated sequence reads data, the results are similar to the simulated SNP array data: the *‘Probabilistic’* approach retained many more markers for mapping at elevated levels of overdispersion. After the initial mapping, ill-fitting markers can be removed resulting in high-quality curated maps.

The markers on the potato SNP array were selected to be of high quality, and therefore, the advantage of using dosage probabilities in this dataset was marginal. We expect that for datasets with a moderate level of overdispersion, the advantages of using probabilistic genotypes will be more evident. Especially in datasets where the level of uncertainty varies between markers, the approach of using discrete dosages will only map the high-quality markers, whereas the probabilistic approach will retain more markers for mapping.

### Application of probabilities for sequencing data

In crop breeding, linkage maps are used to study recombination events along the chromosomes and are in general a pre-requisite for QTL mapping. Although the development of next-generation sequencing allows for physical map assembly, in practice, genetic maps are still important for such studies because, contrary to physical maps, they reflect recombination over the chromosomes, whereas physical sequences reflect base pair distances; linkage maps can therefore more clearly reveal the expected extent of linkage drag around target loci in breeding (Bourke et al. 2018b). Recently published linkage maps of polyploids have primarily been based on discrete genotyping data from SNP arrays. The issue of correctly distinguishing heterozygous dosage classes is potentially more challenging when using read counts of sequencing data instead. Nevertheless, sequencing-based approaches are very attractive for genetic studies of polyploids because of their greater flexibility and lower costs (Spindel et al. 2013) although this would depend on the technology and the sequencing depth.

For polyploids, the large data volume, the frequent occurrence of sequencing and alignment errors, the burden of repetitive and non-informative segments of the genome in sequencing data (Elshire et al. 2011), and the bioinformatics analyses required make the use of sequencing for genotyping purposes more challenging than the analysis of SNP array data. Furthermore, due to the variation in sequencing depth among markers and among individuals, accurate genotyping in polyploids based on read counts is complex (Bilton et al. 2018; Uitdewilligen et al. 2013). Two recently published software packages, updog (Gerard et al. 2018) and polyRAD (Clark et al. 2019) perform genotype calling in terms of discrete dosages and probabilistic dosages based on sequencing data. In both software packages a large amount of missing values for discrete dosages was observed at relatively low read depth (Clark et al. 2019; Gerard et al. 2018). With filtering on missing values, markers or individuals will be filtered out. Relaxing the threshold to save more markers is possible but it also introduces errors. Therefore, given the increasing interest in polyploid genetics and mapping using sequencing-based genotypes, our method will likely become an important addition to the toolbox of researchers investigating the genetics of polyploid species.

## Conclusion

This study explores a method for using dosage probabilities rather than discrete dosages for linkage mapping in polyploid species, implemented in an updated version of MAPpoly (v. 0.2.1.004) and polymapR (v.1.1.0). For high-quality dosage data from SNP arrays, we found that dosage probabilities and discrete dosages perform similarly well. However, the real power and added benefit of probabilistic genotypes are likely to be realized in the use of less-than-optimal SNP arrays and especially in sequence-based genotyping.

### Electronic supplementary material

The file “Supplementary file 1—Figures and Table.pdf” contains supplementary Table 1, and supplementary Ffigures 1–8. The file “Supplementary file 2—Simulations R.pdf” contains the R script used to generate the simulated SNP array data. Below is the link to the electronic supplementary material.

Supplementary file1 (DOCX 703 kb)

Supplementary file2 (DOCX 26 kb)

## Data Availability

We incorporated the use of probabilistic dosage scores in an updated version of the polymapR package (Bourke et al. 2018a). PolymapR is a package for the R language (R Core team 2018), which is available on all major operating systems.

## Appendix 1: Determination of the marker segregation type

To determine the segregation type for each marker, we look at the offspring segregation type and the parental dosages. Probabilistic dosages were used instead of discrete dosages. To obtain the segregation in the progeny, at each locus, we sum the probabilities of each dosage class over the population, rather than summing the individuals per dosage class (as we would do with discrete dosages). As the tails of the probability distributions stretch over the entire dosage ratio or read counts ratio range, also in unexpected dosage classes small probabilities accumulate. A normalization step is performed to minimize the effect of these small estimated probabilities. In the normalization step, we treat the sum of all probability scores per dosage class as 0 if the sum is less than a threshold: 0.05 times the number of individuals divided by (ploidy + 1), the number of dosage classes. For example, with 200 tetraploid individuals, the threshold for the probability sum would be (0.05*200)/(4 + 1) = 2. When the summed probabilities in a dosage class fall below this threshold, all probabilities in that class are set to 0. A subsequent re-normalization step ensures that the normalized probabilities per individual add up to 1 again.

For each segregation type, we consider both the expected and non-expected dosage classes. In a tetraploid species, the expected offspring dosages of an SN marker are 0 and 1, while the unexpected dosage scores are 2, 3 and 4 (double reduction is ignored; previous results of Bourke et al. (2015) show that this hardly affects the estimated maps). For the expected dosage classes, we apply a chi-squared test to the sums of the probabilities per class over the population (similar to the test for discrete dosages, although with non-integer values). For non-segregating markers (e.g., NN markers), we assign a *p*-value of 1. For the unexpected dosage classes, a binomial test is performed to calculate the probability of observing the unexpected dosages, with an expected probability for the error scores of 0.05.

To summarize: we performed a chi-squared test for segregation over the expected classes and a binomial test for the unexpected classes. These values are combined with the parental dosage scores to estimate the marker segregation type and a quality score, as implemented in function checkF1 of package polymapR. Markers with a quality score (*q*_mult_) < 0.05 were rejected. Among rejected markers, the possibility of marker genotype misclassification was also checked, by shifting all dosage classes by either +1 or −1 and re-calculating the aforementioned quality parameters. If a higher quality score resulted, we retained the shifted dosage classes for this marker.

## Appendix 2: Simulation parameters

### Simulation of dosages using PedigreeSim

**Table:**

Number of chromosomes	1
Chromosome length	100 cM
Centromere position	50 cM
Ploidy level	4
Population	F1 (2 parents, 200 offspring)
Inheritance	Polysomic
Marker type	SN, SS, SD, DN, and DD markers were simulated at 1 cM spacings

### Simulation of SNP array intensities

For each marker and each individual, a SNP array produces two signals, X and Y, one for each of the two SNP alleles. These signal intensities were modeled (using the R script in Supplementary file 2) as the summation of two sources of fluorescence intensity: genetic and background.

The genetic part of the signal intensities was obtained in several steps. For each marker, a mean total signal intensity was obtained from a normal distribution with mean 1000 and standard deviation (sd) 0.05, and an allelic bias in the signal responses was obtained from a Beta distribution: Beta (2, 2). For each individual the total signal intensity for that marker was obtained from a normal distribution with the mean as obtained above and a sd obtained from Beta (20, 100). The obtained total signal intensity was then divided over the X and Y signals according to the allele ratio and allelic bias. Finally, overdispersion was added to signals X and Y from a Normal distribution with mean 0 and sd modeled as a proportion of the mean intensity of the marker, ranging from 1 to 6% of the mean. (These values were found empirically to cover the range of dispersions commonly encountered.)

The background part of the signal intensities was constant within a marker. The total background intensity for a marker was obtained from Beta (2,2); this total background was divided over the X and Y signals according to an imbalance factor: *Y*_back_/(*X*_back_ + *Y*_back_) ~ Beta(2,2).

### Simulation of sequence reads using updog

**Table:**

Read depth	Range from 50 to 200
Sequencing error	0.001
Allelic bias (alternative/reference)	0.7
Overdispersion	Range from 0.01 to 0.06, in steps of 0.01

## Appendix 3: Segregation estimation in simulation studies

In fitTetra, dosage probabilities are assigned to each dosage class. In a tetraploid, the possible dosage classes range from 0 to 4 (with probabilities P0–P4). For a SN marker in a tetraploid crop, a few lines of the output of fitTetra are given below. These lines show the results for a single marker, SNP001, in 4 F1 individuals, F1_1, F1_2, F1_3, and F1_4. Maxgeno is the dosage with the highest probability (MaxP).

For the *‘Probabilistic’* approach, the low probabilities shown in gray are discarded and the remaining dosages renormalized to add up to 1 as described in ‘Marker segregation type’.

**Table:**

Marker	Sample	P0	P1	P2	P3	P4	Maxgeno	MaxP
SNP001	F1_1	0.12	0.88	3E-12	1E-03	5E-04	1	0.88
SNP001	F1_2	0.34	0.63	0.03	4E-15	1E-16	1	0.63
SNP001	F1_3	0.97	0.03	2E-05	1E-03	5E-12	0	0.97
SNP001	F1_4	0.01	0.48	0.51	1E-02	1E-08	2	0.51

Under the two approaches used in the simulation study the information from this table, for this marker and these four individuals as used in downstream linkage analysis would be:

**Table:**

Sample	*‘Discrete’*	*babilistic’*
F1_1	m.v.^1^	0: 0.12 1: 0.88
F1_2	m.v	0: 0.34 1: 0.63 2: 0.03
F1_3	Dosage 0	0: 0.97 1: 0.03
F1_4	m.v	0: 0.01 1: 0.48 2: 0.51
Segregation summary	Dosage 0:1:2 segr^2^ 1:0:0	Dosage 0:1:2 segr 1.44:2.02:0.54

^1^ m.v.: missing value. ^2^ segr: the (probability) sums per dosage.

In the *‘Discrete’* approach, the dosages of F1_1, F1_2, and F1_4 become missing because the maximum probability of these individuals at this marker is below the threshold (0.95). The segregation pattern estimated from the discrete or probabilistic data over these four individuals is shown in the bottom row (in the actual simulations the F1 populations were composed of 200 individuals). In the *‘Probabilistic’* approach, all the probability information is used. The segregation pattern is obtained by adding up the probabilities of each dosage class over the whole population. For each locus, the sum of probabilities over all dosage classes is always equal to the number of individuals.
