# Supplementary material for: Using probabilistic genotypes in linkage analysis of polyploids
Source: Theor Appl Genet. 2021 May 25;134(8):2443–57. doi: 10.1007/s00122-021-03834-x (PMC8277618; doi:10.1007/s00122-021-03834-x)
Supplement: Supplementary file 1 — Supplementary file1 (DOCX 703 kb) [file 122_2021_3834_MOESM1_ESM.docx]

## Supplementary Table 1. Expected segregation ratios

Expected segregation ratios for tetraploids according to parental dosages under polysomic inheritance, ignoring double reduction

| **Parental dosages** | **Expected segregation** |
| --- | --- |
| **simplex × nulliplex (SN)^1^** | 1:1 |
| **nulliplex × simplex (NS)** |  |
| **duplex × nulliplex (DN)** | 1:4:1 |
| **nulliplex × duplex (ND)** |  |
| **simplex × simplex (SS)** | 1:2:1 |
| **simplex × triplex (ST)** |  |
| **duplex × simplex (DS)** | 1:5:5:1 |
| **simplex × duplex (SD)** |  |
| **duplex × duplex (DD)** | 1:8:18:8:1 |

^1^ simplex × nulliplex is equivalent to triplex × quadruplex, simplex × quadruplex and triplex × nulliplex for mapping purposes; other listed parental dosages also have equivalent variants

## Supplementary Figure 1. Estimated versus simulated recombination frequency (r) for pairs of SN segregating markers in coupling phase at overdispersion values of 0.01 and 0.02, for the approach using discrete dosages (left) and probabilistic dosages (right)


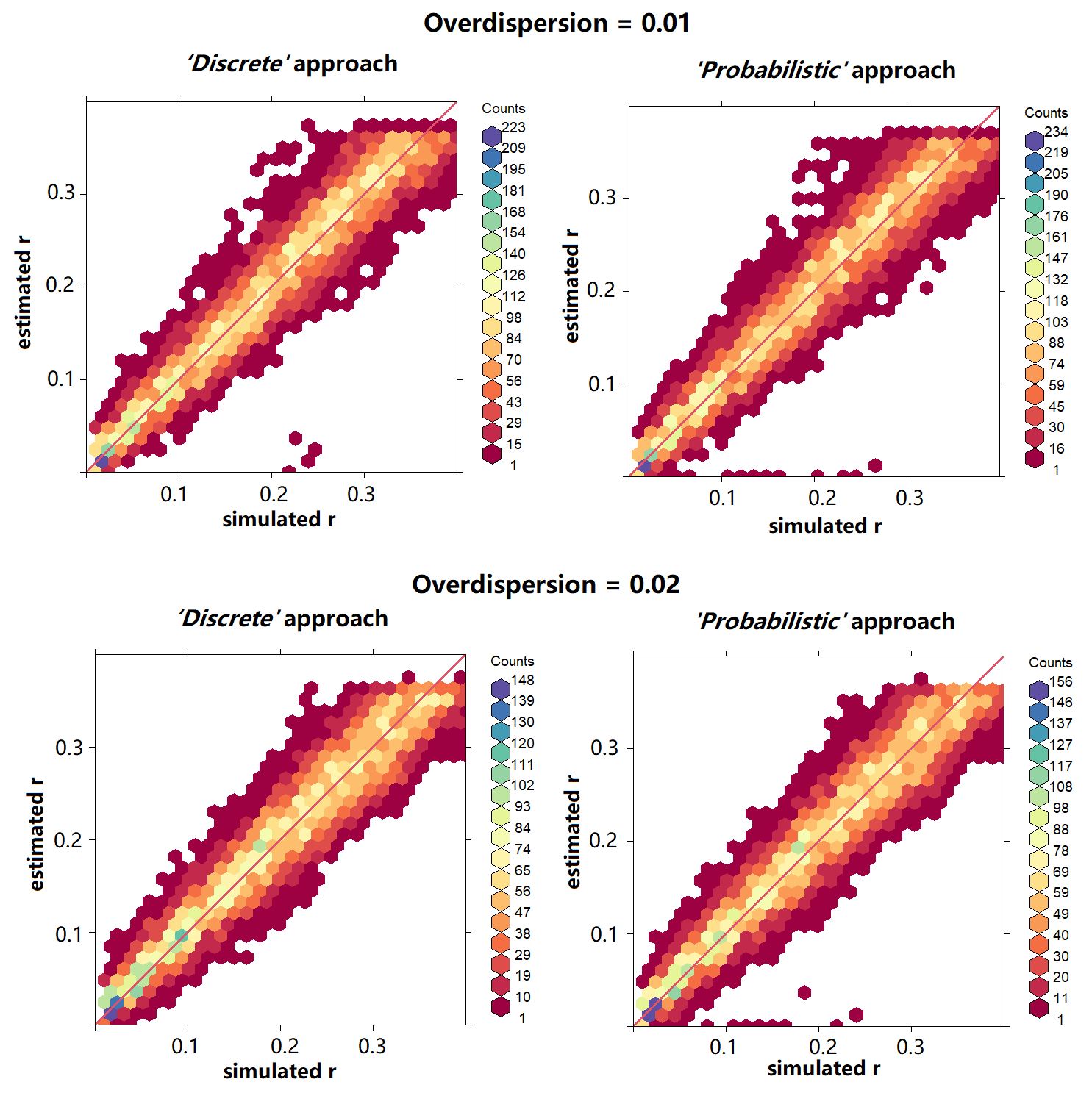


the red line shows y=x. Dots close to the X axis that look like outliers are the wrongly phased markers (simulation was in repulsion phase but estimated to be in coupling phase).

## Supplementary Figure 2. Estimatedversus simulated recombination frequency (r) of pairs of SN segregating markers in coupling phase at overdispersion values of 0.01 – 0.06 for the *‘Probabilistic’* approach


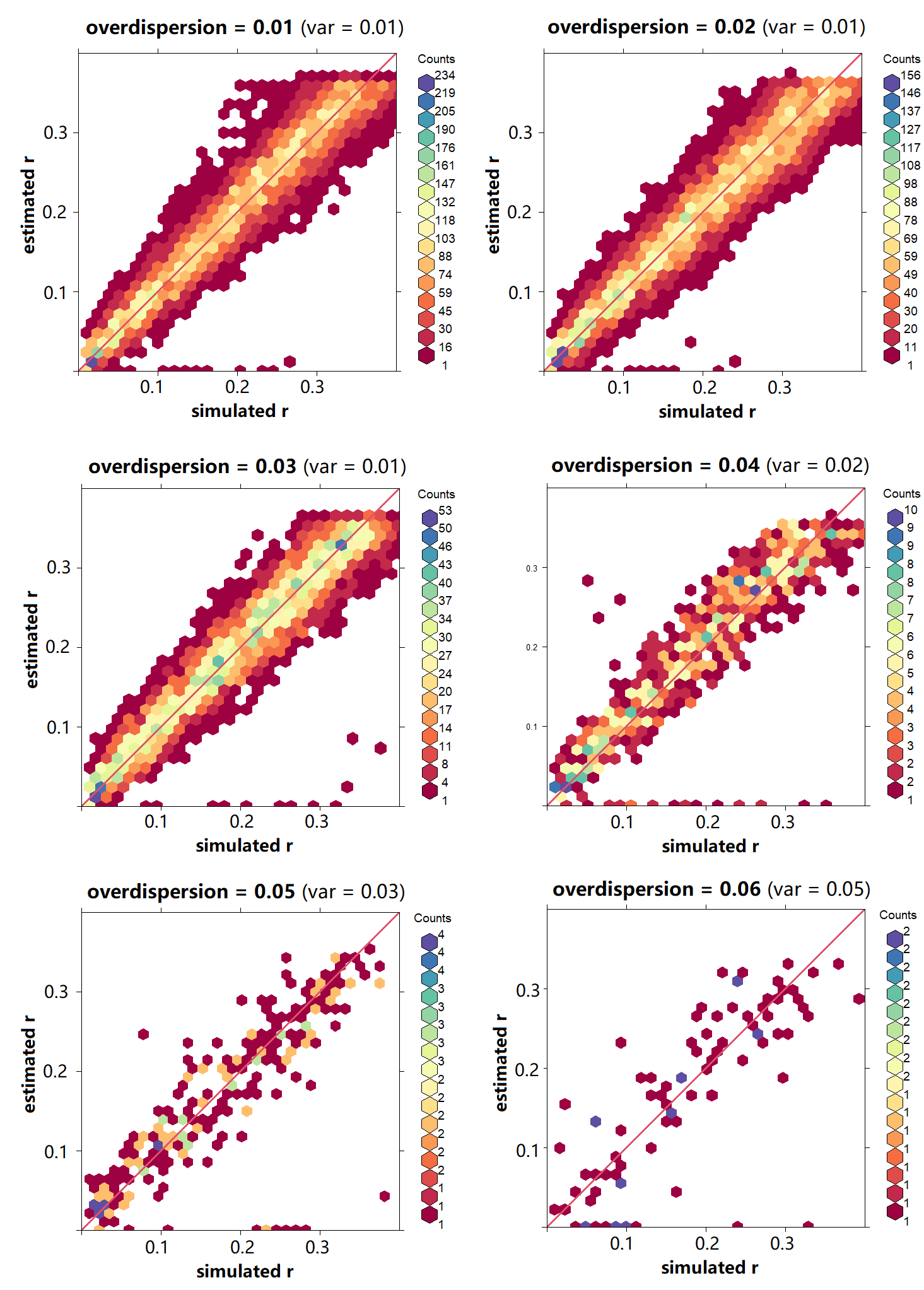


the red line shows y=x. ‘var’ is the squared differences of the simulated r and estimated r. Dots close to X axis that look like outliers are the wrongly phased markers (simulation wass in repulsion phase but estimated to be in coupling phase).

## Supplementary Figure 3. Numbers of markers (totally 505 simulated markers) retained for linkage analysis after determination of the segregation type for the two methods, at different levels of overdispersion


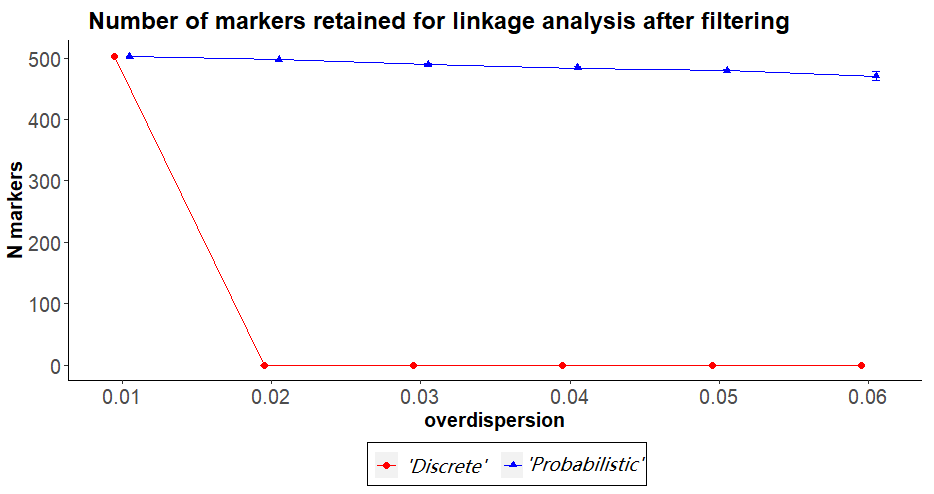


The dots represent the means across 10 simulations for each level of overdispersion setting; standard deviations were smaller than xxx in all cases (not shown). Number of markers retained for linkage analysis after filtering.

## Supplementary Figure 4. The number of SN markers retained for linkage analysis and the regression of estimated r on simulated r for SN-SN pairs for ‘Discrete’ and ‘Probabilistic’ at different levels of overdispsersion in sequence reads simulation


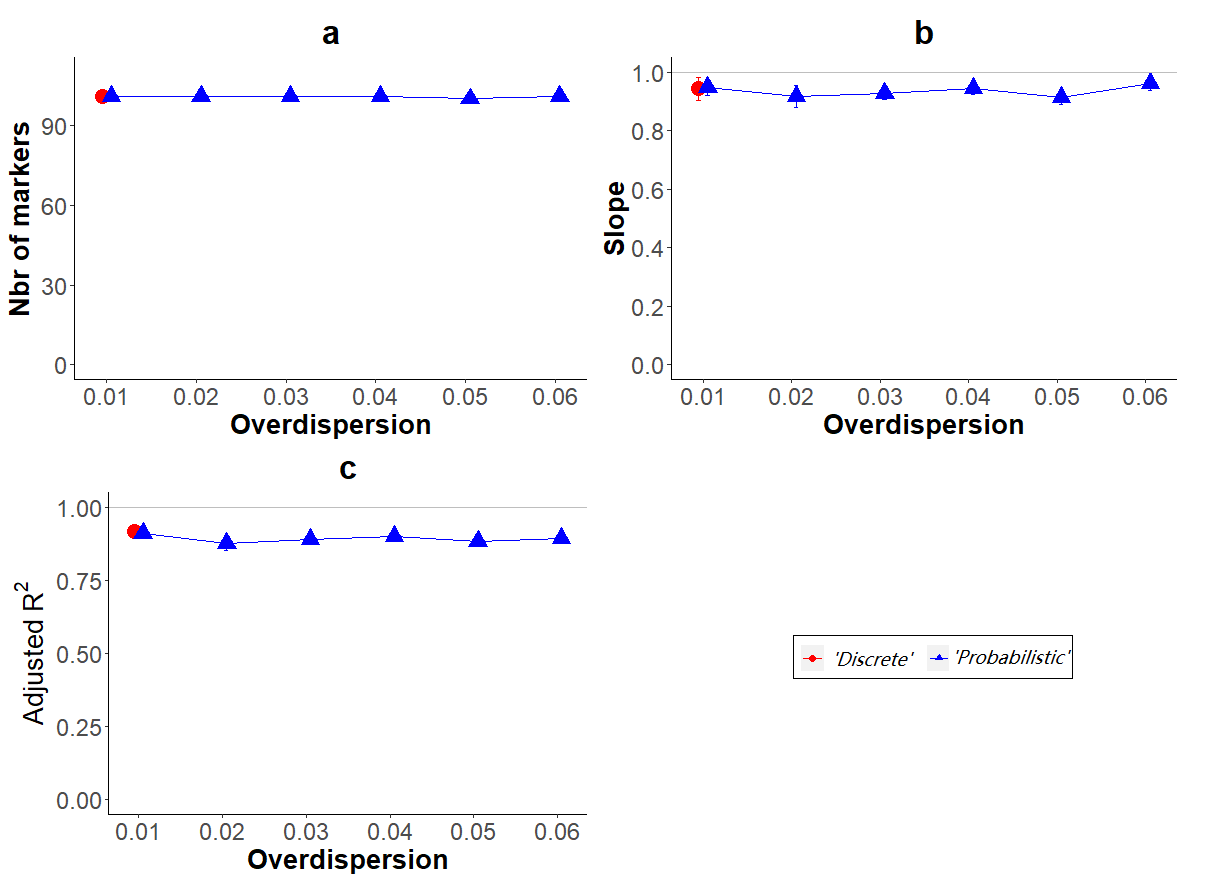


The dots represent the means; error bars represent plus and minus the standard deviation, obtained over all marker pairs in 10 simulations for each overdispersion setting. The grey line shows y = 1: a. the fraction of SN-SN marker pairs retained for linkage analysis (totally simulated 101 SN-SN markers); b. Slope and c. Adjusted R^2^ of the regression of estimated r on simulated r for SN-SN marker pairs

## Supplementary Figure 5. Comparison of map positions for *‘Discrete’* and *‘Probabilistic’* at different levels of overdispersion in sequence reads simulation


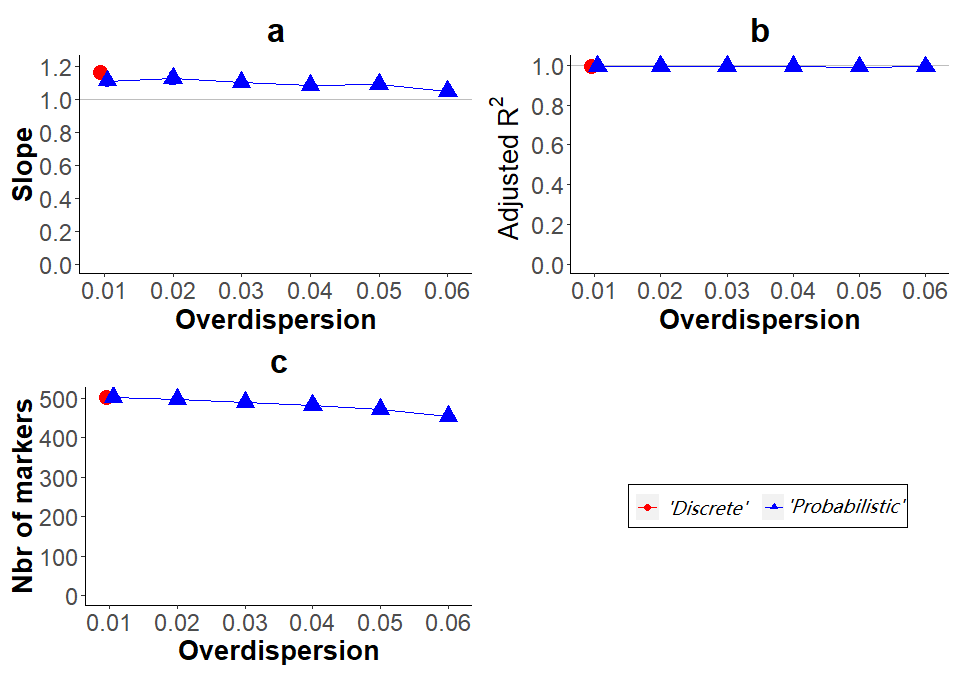


The dots represent the means; error bars represent plus and minus the standard deviation, obtained over all marker pairs in 10 simulations for each overdispersion setting: a. Slope and b. Adjusted R^2^ of the regression of estimated on simulated value of map positions; c. Number of markers mapped

## Supplementary Figure 6. Effect of different threshold values for maximum dosage probability and missing data rate


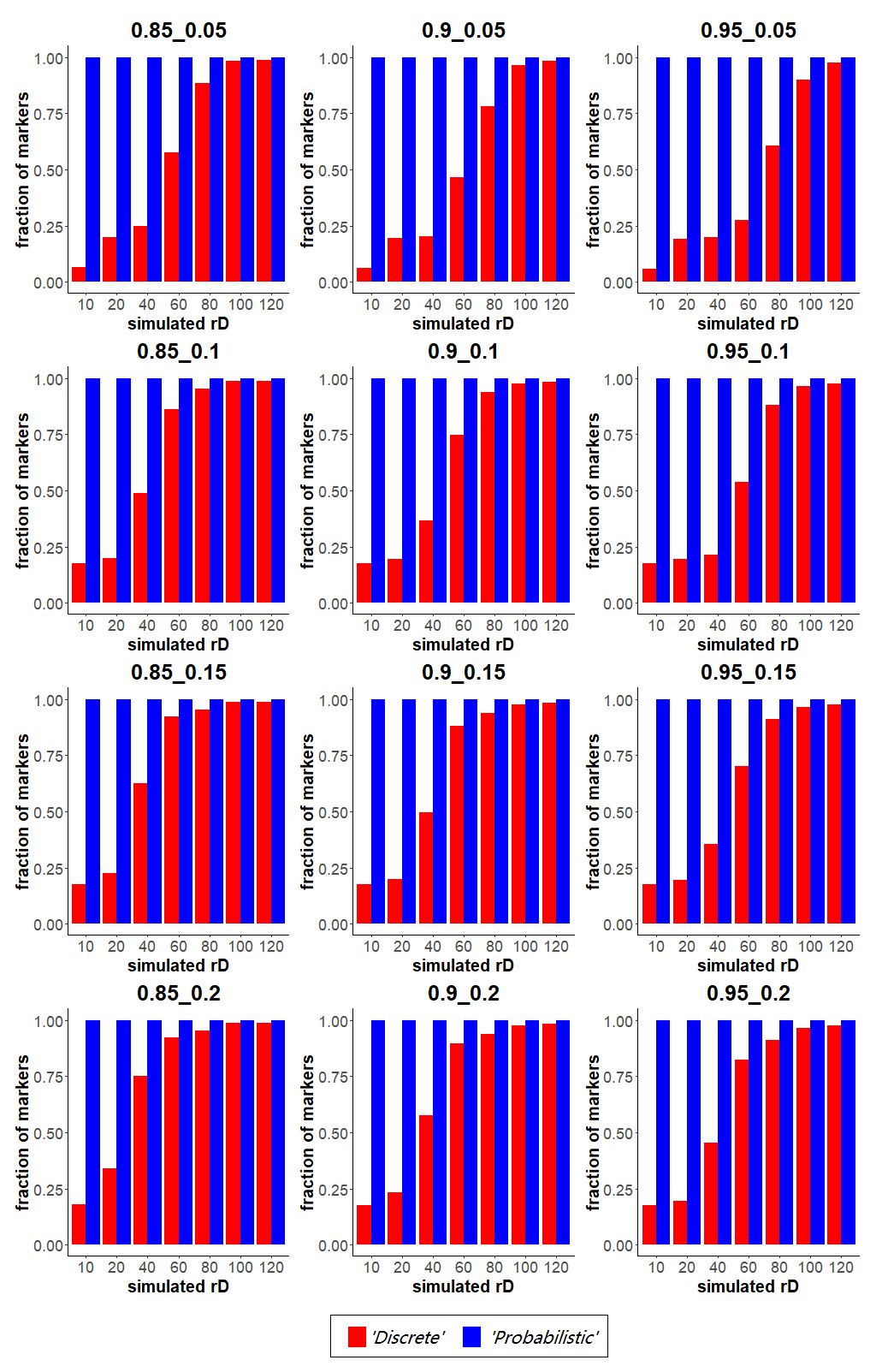


When overdispersion is zero, the fraction of markers retained for linkage analysis in using either the*‘Discrete’* or the *‘Probabilistic’* approach under different threshold values for the maximum fraction of missing values (0.05, 0.1, 0.15, 0.2) and for the maximum dosage probability threshold (0.85, 0.9, 0.95) for a range of simulated read depths.

## Supplementary Figure 7. Additional markers assigned to the potato linkage map using dosage probabilities instead of discrete dosages

**Locations and segregation types of the 562 additional markers**


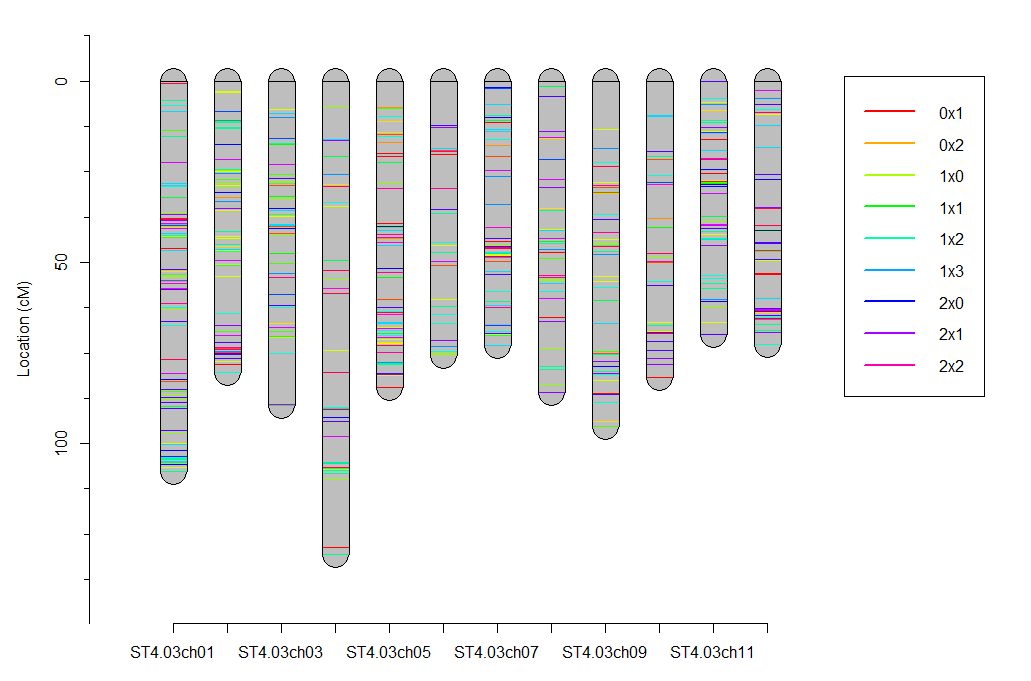


## Supplementary Figure 8. The integrated *‘Probabilistic’* map aligned with the corresponding homolog maps


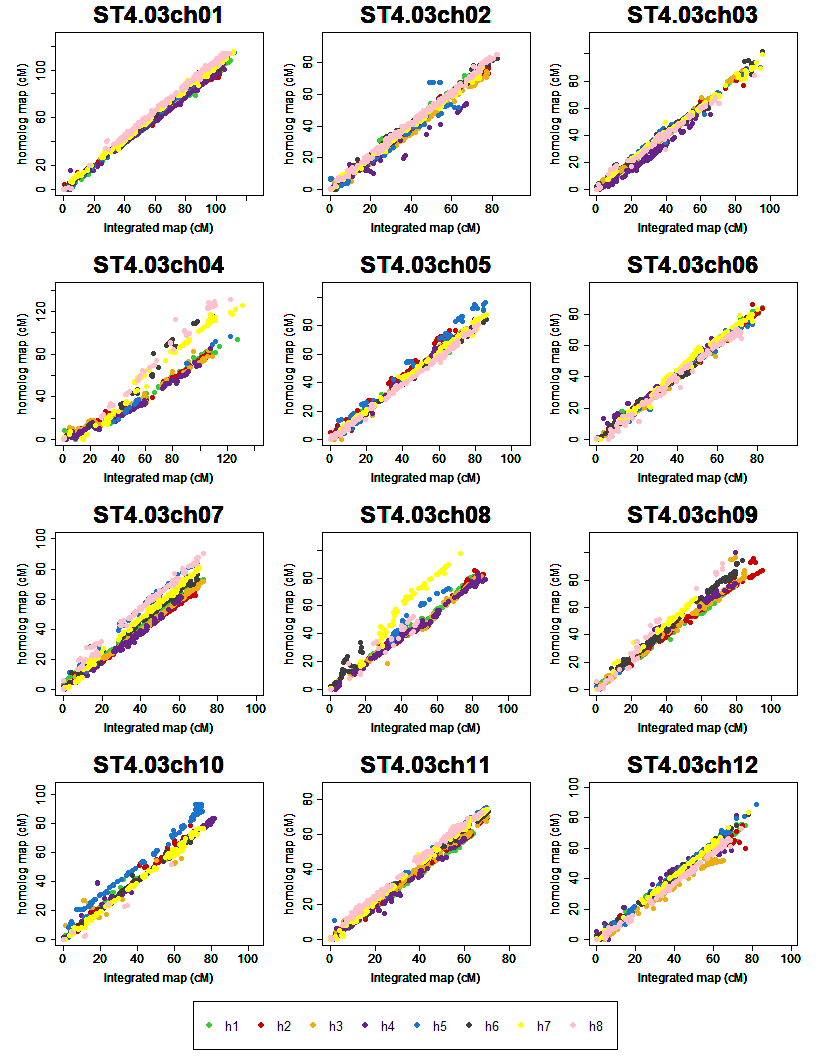


The integrated *‘Probabilistic’* map aligned with the corresponding homolog maps. Colors indicate the eight parental homologs, with h1 – h4 from the female parent and h5 – h8 from the male parent.
