# Supplementary material for: Using probabilistic genotypes in linkage analysis of polyploids
Source: Theor Appl Genet. 2021 May 25;134(8):2443–57. doi: 10.1007/s00122-021-03834-x (PMC8277618; doi:10.1007/s00122-021-03834-x)
Supplement: Supplementary file 2 — Supplementary file2 (DOCX 26 kb) [file 122_2021_3834_MOESM2_ESM.docx]

#' Simulation of SNP array intensities

#'

#' @description \code{SNParraySim} simulates X and Y array intensities, based

#' on a matrix of SNP dosages simulated by PedigreeSim. Individuals of

#' different ploidy can be mixed in the same dosage matrix (e.g. diploid and

#' tetraploid parents of a triploid F1 population). Array intensity (of X or Y)

#' is modelled as the summation of two sources of fluorescence intensity:

#' signal and background (here also called background offset).

#'

#' @param dsg a matrix of allele dosages with markers in rows and individuals

#' in column.

#' @param ploidy a vector indicating the ploidy of each individual.

#' @param avint numeric. It is an overall average intensity

#' @param sd_mrk a number between 0 and 1. It is a coefficient of variation (CV)

#' to define the average Mf of each marker. It will affect the variation of

#' R (R=X+Y) between markers. The default value is 0.05 (5%).

#' @param sd_ind a numeric vector of two elements, defining the parameters shape1

#' and shape2 of a beta distribution, used to sample a coefficient of variation

#' (CV) per marker. This CV will define the sd of Mf values within each marker,

#' affecting in the end the spread of R within markers.

#' @param ale_b a numeric vector of two elements, defining the parameters shape1

#' and shape2 of a beta distribution, used to sample the yunb ratios (allelic bias).

#' @param od numeric, between 0 and 1. It is a coefficient of variation

#' defining the sd for the error term in X and Y simulation, which add extra variation

#' to model.

#' @param bc the parameter shape2 of rbeta (rbeta(n=1000,shape1=2,shape2=10)),

#' used to sample the Roff values (Roff = Roffset/Rsignal).

#' @param bc_XY a numeric vector of two elements, defining the parameters shape1

#' and shape2 of a beta distribution, used to sample Yoff, that is the Y

#' proportion of the background offset intensity.

#' @param seed_number a numeric. Define the seed.

#'

#' @return a list containing X (matrix of X intensity), Y (matrix of Y

#' intensity) and mpar (listing values of yunb, Roff and Yoff per marker)

#' @export

#'

SNParraySim <- function(dsg,

ploidy,

avint = 1000, #overal average intensity

sd_mrk = 0.05, #affect spread of mean intensities (per marker)

sd_ind = c(20,100), #affect spread of intensities within markers

ale_b = c(2,2), #affect XY unbalance (allelic bias) for signal intensity (yunb)

od = 0.02, #affect cluster width (overdispersion)

bc = 10, #affect amount of background offset (Roff)

bc_XY = c(2,2), #affect XY unbalancement for offset

seed_number = NULL) #set a seed for random sampling

{

#### step 1 - convert dosages in expected XY ratios

thr <- sapply(1:ncol(dsg), function(x) {

round(dsg[,x]/ploidy[x],3)

})

#### step 2 - define Mf, a multiplication factor of individual intensity

## Average Mf per marker

# *sd_mrk* is a coefficient of variation (CV), defining the sd of Mf values

if(is.numeric(seed_number)) set.seed(seed_number)

Mf <- rnorm(nrow(dsg), avint, avint*sd_mrk)

Mf[Mf<0] <- 0.1 #to prevent negative Mf values

## Individual variation of Mf (Mf variation within markers)

# Here, we want to define the variation of Mf within each marker, based on a normal distribution.

# Therefore, we need to sample a Coefficient of Variation (CV) for each marker. Since CV values

# ranges from 0 to 1, they are sampled from a beta distribution. A beta distribution is defined by

# the two parameters shape1 and shape2, here called sd_ind [1] and sd_ind [2]. To increase variation,

# that is to make clusters "longer", you need to increase sd_ind [1].

# See the example distributions below:

# hist(rbeta(10000,20,100), xlim = c(0,1))

# hist(rbeta(10000,40,100), xlim = c(0,1))

# hist(rbeta(10000,60,100), xlim = c(0,1))

# hist(rbeta(10000,80,100), xlim = c(0,1))

# The CV is used to calculate the Standard Deviation (SD) of each marker Mf distribution.

if(is.numeric(seed_number)) set.seed(seed_number)

SD <- sapply(Mf, function(x) x * rbeta(1, sd_ind[1], sd_ind[2]))

## full matrix of individual Mf values (with x markers and n individuals)

if(is.numeric(seed_number)) set.seed(seed_number)

Mfi <- t(sapply(1:nrow(dsg), function(x){

sapply(1:ncol(dsg), function(n){

rnorm(1,Mf[x],SD[x])

})

}))

Mfi[Mfi<0] <- 0.1 #to prevent negative Mf values

#### step 3 - sample allelic bias

# yunb is a number between 0 and 1, representing the "fluorescence quantum yield" of Y (Qy),

# relative to Qx. yunb = Qy/(Qx+Qy)

if(is.numeric(seed_number)) set.seed(seed_number)

yunb <- rbeta(length(Mf),ale_b[1],ale_b[2])

#### step 4 - simulate signal intensity of X and Y

# simulate Y

if(is.numeric(seed_number)) set.seed(seed_number)

Y <- t(sapply(1:nrow(thr), function(i){ #per marker

sapply(1:ncol(thr), function(j){ #per individual

Mfi[i,j] * thr[i,j] * yunb[i] + rnorm(1, 0, Mf[i] * od) #add overdispersion

})

}))

colnames(Y) <- colnames(dsg);rownames(Y) <- rownames(dsg)

# simulate X

if(is.numeric(seed_number)) set.seed(seed_number)

X <- t(sapply(1:nrow(thr), function(i){ #per marker

sapply(1:ncol(thr), function(j){ #per individual

Mfi[i,j] * (1-thr[i,j]) * (1-yunb[i]) + rnorm(1, 0, Mf[i] * od) #add overdispersion

})

}))

colnames(X) <- colnames(dsg); rownames(X) <- rownames(dsg)

#### step 5 - background offset

# Roff is a number between 0 and 1, so that Roff = Roffset/Rsignal

if(is.numeric(seed_number)) set.seed(seed_number)

Roff <- rbeta(length(Mf),2,bc)

# Yoff is a number between 0 and 1, so that Yoff = Yoffset/Roffset

if(is.numeric(seed_number)) set.seed(seed_number)

Yoff <- rbeta(length(Mf),bc_XY[1],bc_XY[2])

## add the background offset

Y <- t(sapply(1:nrow(Y), function(i) { #per marker

Y[i,] + mean(Y[i,]+X[i,]) * Roff[i] * Yoff[i]

})) # where mean(Y[i,]+X[i,]) is the mean R signal per marker

X <- t(sapply(1:nrow(X), function(i) {

X[i,] + mean(Y[i,]+X[i,]) * Roff[i] * (1-Yoff[i])

}))

#### step 6 - add balanced background (to prevent negative intensities)

yneg <- apply(Y,1,min); xneg <- apply(X,1,min)

mneg <- apply(cbind(yneg,xneg),1,min) #minimum intensity per marker

mneg[mneg>0] <- 0

Y <- t(sapply(1:nrow(Y), function(i) { #per marker

Y[i,] - mneg[i] + avint*0.1

}))

X <- t(sapply(1:nrow(X), function(i) {

X[i,] - mneg[i] + avint*0.1

}))

#### step 7 - output

mpar <- data.frame(MarkerName=rownames(dsg),

yunb=round(yunb,2),

Roff=round(Roff,2),

Yoff=round(Yoff,2))

output <- list(X=X, Y=Y, mpar=mpar)

return(output)

}
